# Triggers and Anatomical Substrates in the Genesis and Perpetuation of Atrial Fibrillation

**DOI:** 10.2174/157340312803760721

**Published:** 2012-11

**Authors:** Damián Sánchez-Quintana, José Ramón López-Mínguez, Gonzalo Pizarro, Margarita Murillo, José Angel Cabrera

**Affiliations:** 1 Departamento de Anatomía y Biología Celular. Facultad de Medicina. Universidad de Extremadura. Badajoz. Spain; 2 Servicio de Cardiología. Hospital Universitario Infanta Cristina. Badajoz. Spain; 3 Hospital Universitario Quirón-Madrid, European University of Madrid, Madrid, Spain

**Keywords:** Atrial fibrillation, triggers, pulmonary vein, structural remodeling.

## Abstract

The definition of atrial fibrillation (AF) as a functional electrical disorder does not reflect the significant underlying structural abnormalities. Atrial and Pulmonary Vein (PV) muscle sleeve microstructural remodeling is present, and establishes a vulnerable substrate for AF maintenance. In spite of an incomplete understanding of the anatomo-functional basis for AF, current evidence demonstrates that this arrhythmia usually requires a trigger for initiation and a vulnerable electrophysiological and/or anatomical substrate for maintenance. It is still unclear whether the trigger mechanisms include focal enhanced automaticity, triggered activity and/or micro re-entry from myocardial tissue. Initiation of AF can be favored by both parasympathetic and sympathetic stimulation, which also seem to play a role in maintaining AF. Finally, evolving clinical evidence demonstrates that inflammation is associated with new-onset and recurrent AF through a mechanism that possibly involves cellular degeneration, apoptosis, and subsequent atrial fibrosis.

## INTRODUCTION

**Fig. (1):**
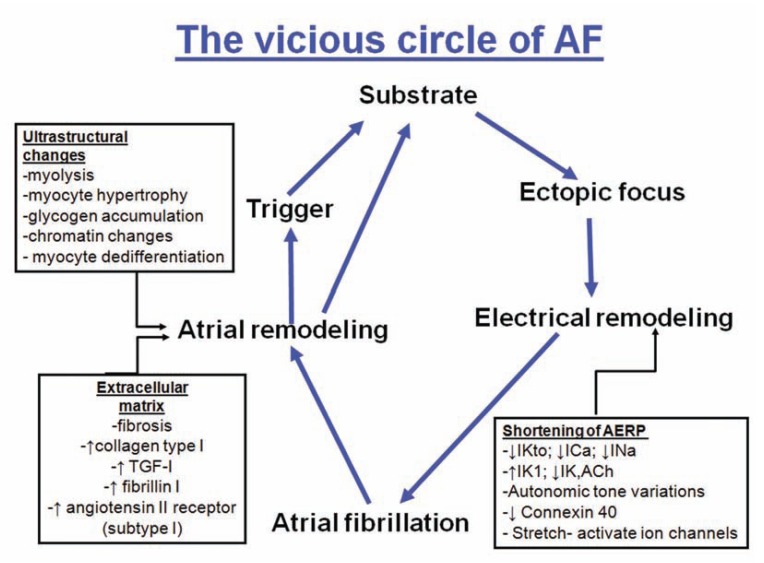
Diagram representing the pathogenesis of the development and maintenance of AF (vicious circle of AF). AERP indicates atrial effective refractory period; IKto, transient outward K+ current; ICa, L-type Ca2+ current; INa, Na+ current; IK1, inward rectifying K+ current; IK, ACh, acetylcholine-regulated K+-current.

There continues to be a lack of understanding of the pathogenesis of atrial fibrillation (AF). Current evidence suggests that the pathogenesis of AF is multifactorial, because this arrhythmia may accompany a variety of pathological conditions (mitral valve disease, hyperthyroidism, hypertension, coronary artery disease, etc.) and may also occur in the normal heart, a condition known as ‘lone AF’ [1]. It has been shown that most of the paroxysmal AF is because of triggers originating from pulmonary veins (PVs) and non-PV sites [2,3]. However, ectopic foci may not always be necessary for the initiation and maintenance of AF. In the early work by Allessie *et al*. [4] decreased wavelength alone (product of refractory period and conduction velocity) allowed for the maintenance of several simultaneous re-entry circuits leading to the development of AF. The concept of electrical remodeling, along with the associated alterations in the number and function of ion channels, has been implicated in the progressive nature of the disease and the notion that “AF begets AF” [5]. The presence of a susceptible atrial anatomical substrate with areas of conduction block, causing spatial dissociation of the wavelets and promoting re-entry, has been implicated in the perpetuation of the arrhythmia [6] (Fig. **1**),the so called chronic AF (persistent or permanent). In chronic AF, atrial remodeling increases the complexity of wave propagation, leads to a multiplication of sites with maximal dominant frequency (known as rotors) and shifts their location away from the PV region into the left atrium (LA) and/or right atrium. However, it remains unclear whether this structural remodeling, with features of interstitial fibrosis and myolysis, or an increase in autonomic tone are pro-fibrillatory factors; or whether AF can be induced or produced simply as a feature of older age or underlying heart disease [7]. 

## TRIGGERS OF ATRIAL FIBRILLATION

### A Pulmonary Veins 

**Fig. (2):**
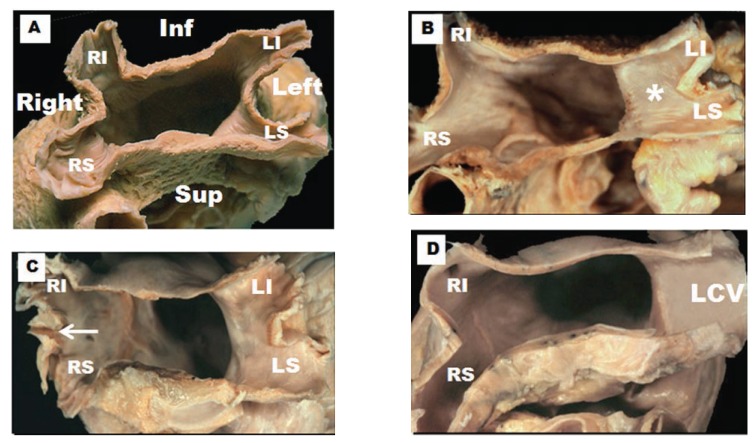
Four hearts specimens with the roof of the left atrium removed and the observer looking towards the mitral orifice. Note the variable pulmonary vein and their venoatrial junctions anatomy between specimens. (**A**) The arrangement of four pulmonary venous orifices. (**B**) Left pulmonary veins form a short vestibule (asterisks) or funnel-like common vein before opening left atrium. (**C**) Three orifices on the right side (arrow). (**D**) A common vein on the left side. RI, right inferior pulmonary vein; RS, right superior pulmonary vein; LI, left inferior pulmonary vein; LS, left superior pulmonary vein; LCV, left common vein.

In recent times various non-invasive imaging techniques such as magnetic resonance imaging and multidetector computed tomography have shown a variable pulmonary vein (PV) anatomy among patients. An example of this variability is the number of PVs: some patients have 5 distinct ostia while others have common trunks. A common left or right pulmonary vein is seen in 25% [8,9] being more frequently on the left than the right PVs. The existence of “extra” PVs, the most common is a separate right middle PV draining the middle lobe of the lung, is another common finding [10] present in up to 26% of patients [11]. The ostial diameter of this right middle PV is smaller than other veins (mean, 9.9 ± 1.9 mm versus 21.6 ± 7.5 mm). Our anatomical study on a series of 35 heart specimens (Fig. **2**) found the classic arrangement of 4 orifices in 74%, with 31% of these in the setting of a short vestibule or funnel-like common vein. Five venous orifices were found in 17%, and the remaining 9% had a common vein on the left or right side [12]. In the classic pattern, the right superior PV passes behind the junction between the right atrium and the superior caval vein, whereas the right inferior PV passes behind the intercaval area. The orifices of the right PVs are directly adjacent to the plane of the atrial septum.

It is important to establish the fact that, as the group of Michel Haïssaguerre^2^ demonstrated, the LA is electrically connected to the PVs, by atrial muscle sleeves extending from the LA myocardium to the PV. Most of the focal triggers (80%) are located in this myocardium. They can create propagating wavelets, which in the presence of reduced refractory period and/or conduction velocity, may lead to re-entrant circuits and AF. These authors noted an unequal distribution of PV ectopy (i.e., 31 foci in left superior PV, 17 foci in right superior PV, 11 foci in left inferior PV, and 6 foci in right inferior PV) [2]. At present, the electrical isolation of all PVs is the key strategy of any transcatheter ablation procedure that attempts to cure AF. In laboratories with extensive experience, this procedure is accompanied by a relatively high success (> 85%) in patients with paroxysmal AF [13-15]. Some centers have reported a 65% success rate in patients with chronic AF (persistent or permanent), but this usually requires, in addition to pulmonary vein isolation, other procedures such as biatrial defragmentation, and anatomical linear ablation with the endpoint of AF termination [16]. 

**Fig. (3):**
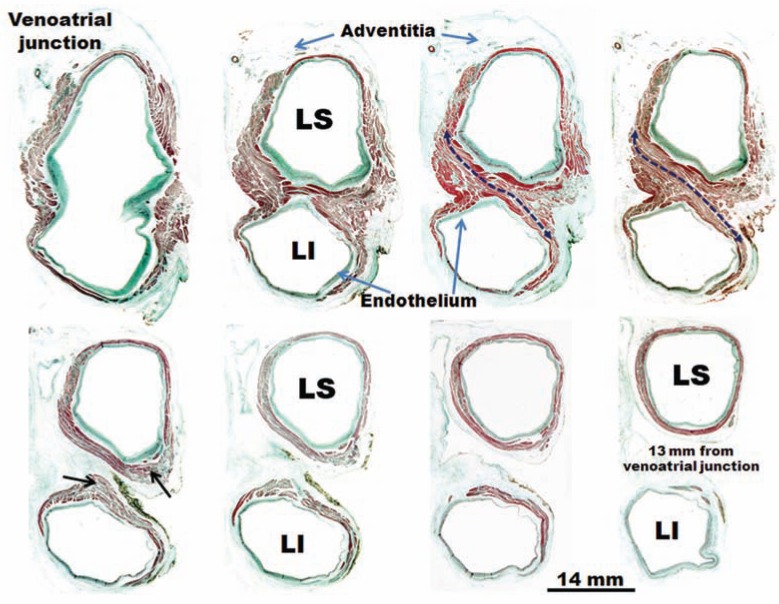
Histological cross-sections with trichrome staining through eight sets of human left pulmonary veins showing the variations in circumferential arrangement of the myocardial sleeves. Note the myocardial sleeve completely surrounding the superior vein and extending beyond 10 mm from the venoatrial junction in a male specimen. The sleeves are thicker in the sectors close to adjacent veins (arrows). Note myocardial strands/ bridges crossing the interpulmonary isthmus or carina between superior and inferior venous sleeves at the level of the venoatrial junction (broken arrow). LI, left inferior pulmonary vein; LS, left superior pulmonary vein;

**Fig. (4):**
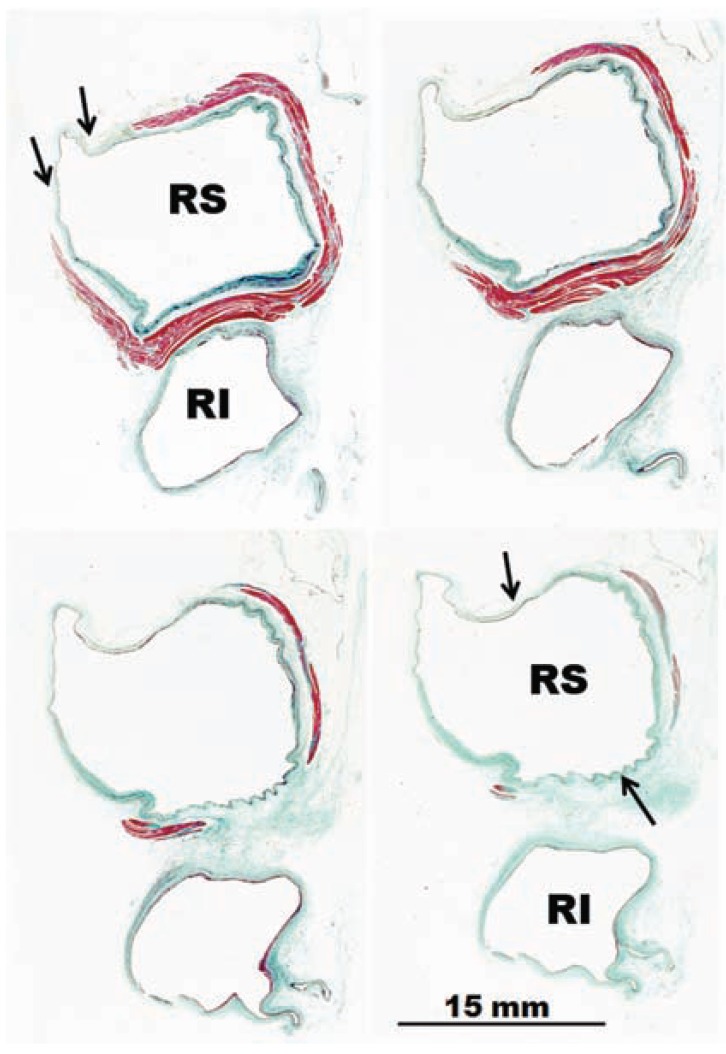
Histological cross-sections with trichrome staining through four sets of human right pulmonary veins. In contrast to figure 3, there is no sleeve surrounding the inferior vena from the venoatrial junction to 10 mm. Note the incomplete encirclement of the sleeve at the level of the right superior pulmonary vein (arrows). RI, right inferior pulmonary vein; RS, right superior pulmonary vein.

**Fig. (5):**
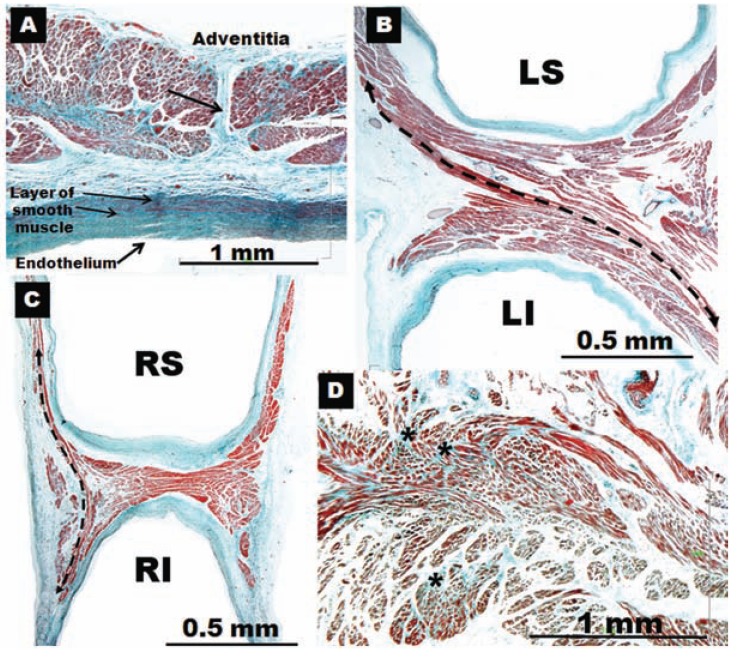
Four histological cross-sections with trichrome staining. Note in (**A**) gap in the myocardial sleeve composed of fibrous tissue (arrow) extending between the adventitia and a media layer of smooth muscle next to endothelium. (**B**) Interpulmonary myocardial connection at the venoatrial junction (broken arrow). (**C**) Interpulmonary myocardial connection at the ipsilateral anterior wall of adjacent right pulmonary veins (broken arrow). (**D**) A small focus of fiber disarray and interstitial fibrosis (asterisks) between the left atrium and left superior pulmonary veins. RI, right inferior pulmonary vein; RS, right superior pulmonary vein; LI, left inferior pulmonary vein; LS, left superior pulmonary vein; LCV, left common vein.

Knowledge of the anatomy of PVs is important in understanding their electrical properties. Early work by Nathan and Eliakim [17] described the presence of muscular sleeves continuing from the LA into the PV with a mean extent of 13mm and a maximal extent of 25mm. These sleeves had variable lengths and were better developed in the upper than in the lower veins. We have demonstrated the variable anatomy of the PVs myocardium [18,19]; although the ostia of the veins were ellipsoid with a similar diameter (approx. 1 ± 0.5cm, range 8–20 mm), the length of the myocardial sleeves had a distinctive distribution. The longest sleeves were over the superior veins, with the left PV being longer (1.1 ± 0.3cm) than the right (0.9 ± 0.3cm). Our histological studies [18,19] showed that the walls of the PVs were composed of a thin endothelium, a media of smooth muscle and a thick outer fibrous adventitia (Figs. **3** and **4**). The transition from atrial to venous walls was gradual as the myocardial sleeves from the LA overlapped with the smooth muscle of the venous wall. The myocardial sleeves lied external to the venous media and internal to the epicardium/adventitia. They were thickest at the venoatrial junction (mean 1.1mm) and thinned out distally. Furthermore, the thickness of the sleeves was not uniform, with the inferior walls of the superior veins and the superior walls of the inferior veins having the thicker sleeves (Fig. **3**). Importantly, we were able to show two structural features in hearts without heart disease: i) that throughout the PV, and even at the venoatrial junction, there were gaps in the myocardial sleeves that were mainly composed of fibrous tissue [19]. These gaps create discontinuities between groups of myocytes (Fig. **5**).ii) interpulmonary myocardial connections are common, occurring in 83% (15 of 18 specimens) of the hearts examined (Fig. **5**) [20]. At the venoatrial junction, 27% of the myocardial strands/bridges crossed the interpulmonary isthmus or carina at the subepicardium; 53% at the subendocardium and 20% both at the epi and subendocardial aspects [20]. Other myocardial strands connected the ipsilateral anterior and posterior walls of adjacent PVs in 40% of the hearts (Fig. **5**)[20]. 

The arrangement of the myocyte bundles within the sleeves was found to be rather complex. In contrast to previous reports [17], there appeared to be a meshlike arrangement of muscle fascicles, made up of circular-orientated bundles that interconnected with bundles that ran in a longitudinal orientation [19] (Fig. **5**). We have suggested that such an arrangement leads to anisotropic conduction between the bundles, which can act in itself as a focal trigger/micro re-entry. Patchy areas of fibrosis, which were also detected, may take an active part in the "AF initiation" role of the PVs [19]. Interestingly, no correlation was seen between patient age and histological appearance. Another important anatomical feature is that these myocardial connections may be the anatomical substrate for electrical links between different PVs. This may have clinical implications for local PV disconnection when attempting AF radiofrequency catheter ablation [20]. Importantly, the convoluted, interwoven muscle bundles as described in the carina between veins may also make it more likely to reconnect PVs with either end of the muscle bundle. This anatomical feature would explain why frequent triggers may result in local re-entry and actually precipitate AF [21,22], consistent with clinical observations.

**Fig. (6):**
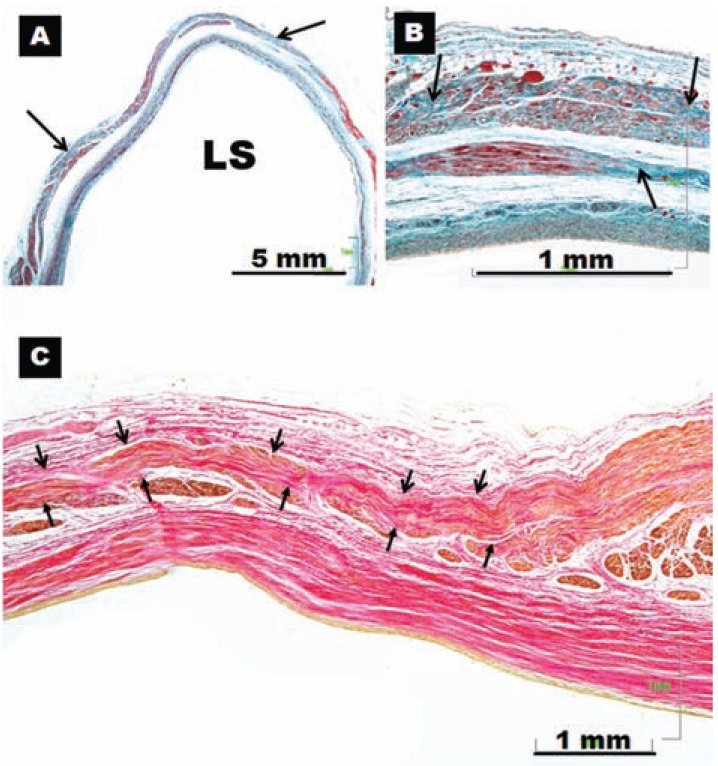
(**A**) and (**B**) Histological cross-sections with trichrome staining in two different specimens. Note the high degree of fibrosis (arrows) of the myocardial sleeve in the left superior pulmonary vein of a specimen with atrial fibrillation. (**C**) Longitudinal section with van Gieson staining. Note the interstitial fibrosis and scarred peripheral projection of a sleeve (between arrows) acquiring a serpentine shape of a patient with atrial fibrillation. LS, left superior pulmonary vein.

Several studies have been designed to investigate and to compare the pathology of the PVs in patients with and without AF [23-27]. Although patients with AF have a higher number of pathological alterations, anatomical findings are similar. We should note that specialized cells, particularly node-like cells, were not examined in these studies. Hassink *et al*. [23] compared the PVs of 6 patients with a history of permanent AF with 14 patients with no history of AF. The histological analysis revealed expansion of atrial myocardium into 89% of all PVs. Prevalence of myocardial extension was significantly higher in veins of patients with permanent AF. Other significant histological differences between both groups were a higher frequency of discontinuity and hypertrophy and a higher degree of fibrosis of the atrial myocardium extended in the PVs of patients with AF (Fig. **6**). Saito and colleagues [24] compared the myocardial sleeves of 22 patients with a history of paroxysmal AF with 17 patients with no history of AF. There was a high incidence of structural heart disease in both groups of patients. However, Saito and his colleagues reported no discontinuity of the extended atrial myocardium. Tagawa *et al*. [25] demonstrated that, although the superior PVs were similar between patients with and without a history of AF, the myocardial sleeves on the inferior PVs were longer in those patients with AF. Morphological details, such as the presence of hypertrophic myocytes and discontinuous myocardium, were not reported in this study. Kholova *et al*. [26] showed that the length and thickness of the muscular sleeve of the left superior PV was greater in AF patients compared with controls whilst the differences for the right superior PV did not quite reach statistical significance. Finally, Steiner *et al*. [27] demonstrated that isolated atrial amyloid was deposited not only in the atrial walls but also in the myocardial sleeves of the PVs; it was present in 55% of sleeves in a group of 100 hearts from patients both with and without AF. Generally isolated atrial amyloid may be deposited in the heart as a manifestation of aging. The deposits were more marked in the central parts of the sleeves than at their periphery. The superior PVs contained more amyloid than the inferior ones, and the right PVs were more affected than the left. The amyloid was present in 76% of patients with AF, while the value was 60% in patients in sinus rhythm. The differences, however, did not reach statistical significance. In these autopsy studies, the presence of fibrosis of the myocardial sleeves was examined and correlated both with and without AF but did not correlate with the patient's age [19]. The patients with AF had significantly more marked fibrosis than those in sinus rhythm. Fibrosis was more severe in the peripheral parts of the sleeves, with fingerlike projections often totally sclerotic (Fig. **6**). The character of this atrial fibrosis differs from postnecrotic myocardial scars in ischemic heart disease. Some authors [27] have suggested that fibrosis presumably occurs more in the peripheral parts of the sleeves, which are located at the very periphery of the coronary circulation, because they are in a state of chronic hypoxia with consequent progressive degeneration of the myocardium resulting in fibrosis. The genesis of the scarring is, therefore, probably not postnecrotic but degenerative [27]. A concept promoted by Spach and Boineau [28] who argued that development of extensive collagenous septa induced nonuniform anisotropic electrical propagation that could result in micro re-entry, and thus form a basis for the high incidence of atrial tachyarrhythmias in older people.

Several years ago, Pérez-Lugones *et al*. identified sinus node-like cells (P cells) within human PVs that were associated with AF [29]. These myocardial cells were identified under light microscopy by their pale cytoplasm and their positive response to periodic acid-Schiff (PAS) staining in 4 of the 5 autopsy subjects. Electron microscopy confirmed the presence of these P cells, which are probably the site of nodal impulses and consist of myocardial cells, small and round or ovoid, with an empty-appearing cytoplasm, sparse myofibrils and small mitochondria. Transitional cells and Purkinje cells were also found in the pulmonary veins of these cases. Reports in the literature exist describing a close spatial relationship between PAS-positive cells and sympathetic nerves near the sites of PV muscle sleeve ectopy in canine models of AF [30,31]. Whether these PAS-positive cardiomyocytes have pacemaker currents or are a potential source of automaticity remains unknown. Currently, we morphologically distinguish cardiac conduction tissues on the basis of three histological criteria proposed by Mönckeberg [32] and Aschoff [33] in 1910: i) the cells comprising the proposed tracts should be histologically distinct from their neighbors; ii) it should be possible to follow them through serial sections; and iii) they should be separated from the remainder non-specialized adjacent working myocardium by insulating sheaths of fibrous tissue. Nodal and transitional cells satisfy two of these criteria [34], while PAS-positive myocytes of AF patients, being histologically distinct, satisfy only one criterion. Later, other authors as Morel *et al*. [35] and Gherghiceanu *et al*. [36] demonstrated the presence of interstitial Cajal-like cells within the muscular sleeves of PVs. However, neither of these studies characterized the electrophysiological properties of these interstitial Cajal-like cells or explored mechanisms by which they may contribute to atrial arrhythmias. More recently, Levin *et al*. [37] characterized a novel, melanocyte-like cell population in the heart and PVs that contribute to atrial arrhythmogenic triggers in mice. These murine cardiac melanocyte-like cells are electrically excitable, and express adrenergic and muscarinic receptors. However, the exact distribution of cardiac melanocytes in the human heart remains to be confirmed, as well as their contribution to AF. In either case, these studies represent potential pacemaking activity of non-cardiomyocytes within PVs which may be integral to atrial arrhythmogenesis.

It has been known for some time that the atria and PVs contain enriched autonomic innervation, and that autonomic output is a significant contributing factor to the initiation and maintenance of atrial arrhythmias [38-40]. Several animal studies and human biopsies taken from the PV-LA junction demonstrated the important role of the autonomic nervous system in AF initiation and maintenance [41,42]. In fact, sympathovagal imbalance causes anisotropic changes in the action potential durations and refractory periods of myocytes at the PVs sleeves and atria [41]. Due to this sympathovagal discharge, action potential duration might be shortened, promoting re-entrant excitation. While targeting autonomic cardiac ganglia alone did not prevent long-term AF recurrences [43], it has been shown to improve the cure rate, when used as an adjunct to surgical PV isolation [44]. The presence of innervation within the myocardium of the LA suggests that non-transmural lesions could have a clinical impact during catheter ablation. However, the autonomic nervous system elements are mainly present in the fat pads, on the epicardial surface of the LA wall, and therefore transmural lesions are needed to denervate the atria [45]. The contribution of the neural inputs to the ablation areas points to a complex interplay of anatomical and electrophysiologycal substrates for the genesis and recurrence of AF.

### B Non-PV Ectopic Triggers

Non-PV ectopic triggers that initiate paroxysmal AF have an incidence of 28% and may arise from the superior caval vein (SCV) (37%), left atrial posterior wall (38.3%), terminal crest (TC) (3.7%), coronary sinus (CS) (1.4%), oblique vein/ligament of Marshall (8.2%), and interatrial septum (IAS) (1.4%) [46-51]. The predominant non-PV triggering sites have a slow pacemaker-like current prior to the actual swift depolarization cellular. The triggered activity of the non-PV triggers could also be involved in the onset and perpetuation of AF. Previous studies have documented triggered activity with delayed afterdepolarizations in the sites mentioned above. Furthermore the left atrial appendage appears to be responsible for triggering AF in 27% of patients presenting for repeat procedures of catheter ablation [52]_._

#### B1 Superior Caval Vein (SCV)

**Fig. (7):**
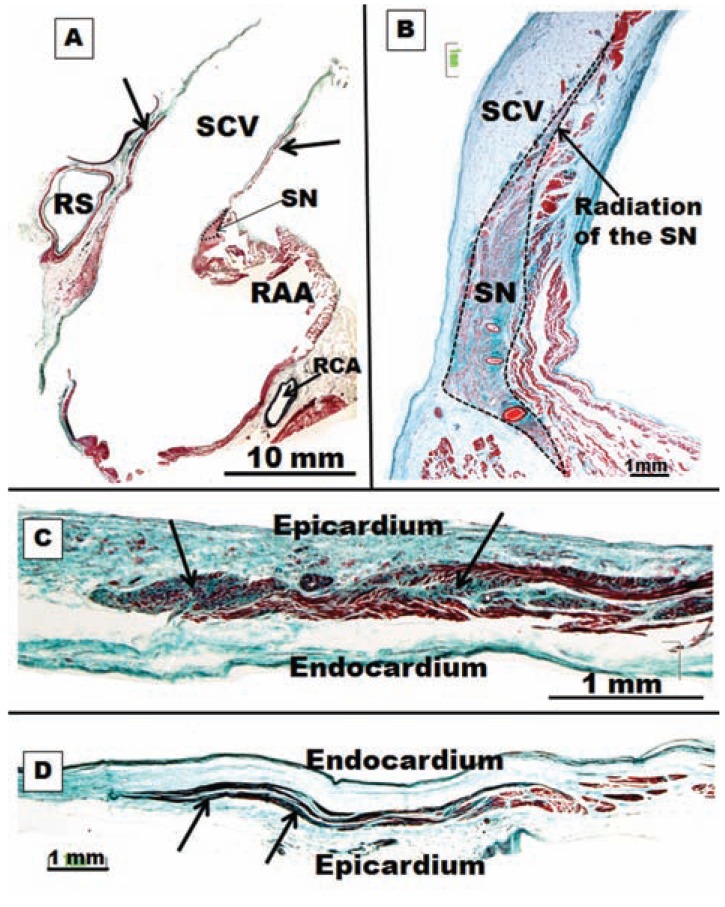
(**A**) Low-magnification photomicrograph of superior caval vein (SCV), myocardial sleeves (arrows), right atrial appendage (RAA) and sinus node (SN). RS, right superior pulmonary vein, RCA, right coronary artery (**B**) Sagittal section of the superior caval vein with trichrome staining. Note the overall architecture of the sinus node (SN) with a radiation located in the superior caval vein (SCV). (**C**) Histological cross-sections of superior caval vein with trichrome stain showing degenerative changes in a myocardial sleeve in a specimen without history of atrial fibrillation. These changes were more marked in the periphery parts of the myocardial sleeve. (**D**) The same structural features in C but in a specimen with history of atrial fibrillation.

The proximal SCV contains myocardium that connects to the right atrium (Fig. **7**), and thus atrial excitation or sinus node impulse can propagate into the SCV [53]. SCV cardiomyocytes were found to have pacemaker activity, and the enhanced automaticity and afterdepolarization play a role in the arrhythmogenic activity of SCV [54]. In the structural study by Kholová and Kautzner [55], morphological and morphometric characteristics of working atrial myocardial extensions onto caval veins (CVs) were compared in subjects with and without a history of AF. Their major findings can be summarized as follows: (1) working myocardial extensions were revealed in 76% of CVs and were equally frequent in the SCV and the inferior caval vein (ICV); (2) these extensions were localized on the subepicardium in all subjects (Fig. **7**), and their maximum length reached up to 61 mm and maximum thickness up to 4 mm; (3) myocardial fibers in the sleeves were arranged predominantly circularly to the long axis of the vein and were frequently discontinuous; (4) no specialized conduction cells were observed; (5) degenerative changes were described in approximately one third of all myocardial extensions; (6) atriocaval junction assessment revealed a predominantly continuous pattern in the SCV in few subjects, whereas discontinuity was present in ICV; and (7) no major differences in characteristics of myocardial sleeves around the CVs were found between patients with and without a history of AF. 

Other authors [56] have noted, using gadolinium-enhanced magnetic resonance angiography with three dimensional reconstruction, that the patients with AF initiated by SCV ectopic beats exhibited a more eccentric structure of the second part or distal part of the SCV as compared to the control group. In fact all ectopic beats initiating AF were located in the second part of the SCV. Furthermore, these patients had a larger SCV volume, left atrial volume and PV size, and had more eccentric PV ostia than controls. However, an animal study examining the canine SCV showed that the proximal segment of the SVC is a complicated structure comprising of a variable size and assembly of individual cardiomyocytes and an irregular distribution of gap junctions and expression of their component connexins. These features may potentially contribute to its arrhythmogenicity [57]. Previous embryological studies have demonstrated that the sinus node (SN) is derived from the sinus venosus, and that other remnants of the embryonic sinus venosus are present in several areas of the mammalian heart, including the musculature of the SCV and an area embedded in the proximal terminal groove [58-59].

Our studies [60] showed that in 74% of the specimens there were 1–10 radiations from the sinus node (0.2–2 mm long), entering into the atrial wall and extending superiorly towards the SVC, inferiorly towards the subepicardium, and intramurally into the ordinary myocardium of the terminal crest or intercaval area. These radiations of the sinus node were histologically discrete, but they were not insulated from the remaining atrial myocardium. Sometimes, the overall architecture of the sinus node with its radiations was located towards the SCV and, as in the PVs, we observed degenerative changes in the working atrial myocardial extensions onto SCV in subjects with and without a history of AF (Fig. **7**). These changes were more marked at the periphery parts of the working myocardial extensions than at the proximal segment of the SVC (Fig. **7**). 

#### B2 Oblique Vein/Ligament of Marshall

**Fig. (8):**
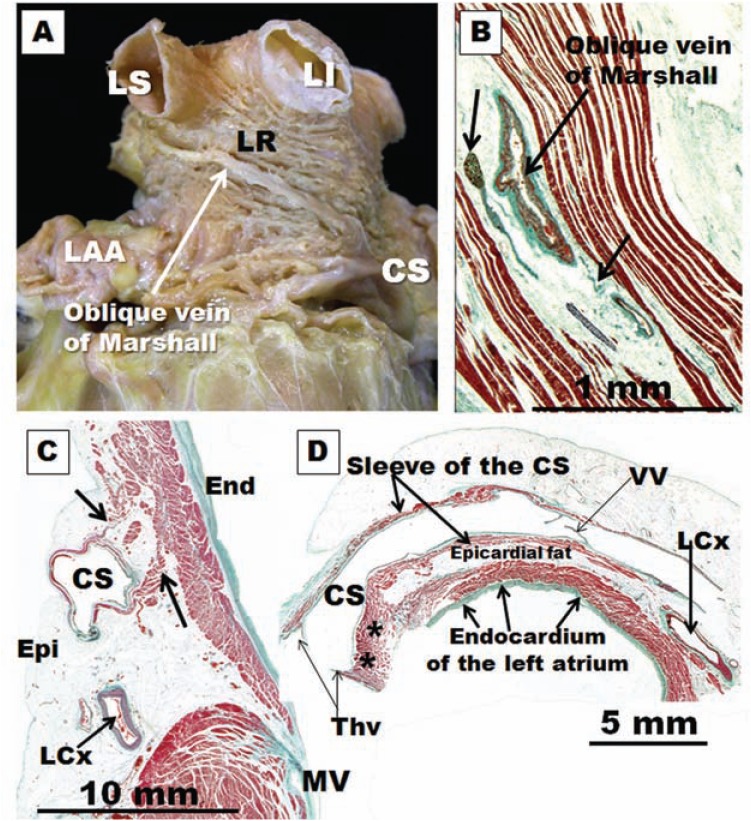
(**A**)Dissection of the left lateral wall of the left atrium to show the myofiber arrangement in the subepicardium of the lateral ridge (LR). Note the vein of Marshall in relationship with LS, left superior; LI, left inferior pulmonary veins. CS, coronary sinus; LAA, left atrial appendage. (**B**) Sagittal section showing the oblique vein of Marshall and the arrows indicating ganglion and nerve bundles in the vicinity of the vein. (**C**) Histological section through the left atrioventricular junction shows the coronary sinus (CS) and circumflex artery (LCx) . The CS is surrounded by a sleeve of muscle. There is muscular continuity (arrows) between the sleeve and posterior left atrial wall. MV, mitral valve. (**D**) Histological cross-sections (Masson trichrome stain) through the coronary sinus (CS) demonstrate the coronary sinus-left atrium muscle connection (asterisks) at the distal end of the coronary sinus. The rest of the myocardial sleeve of the CS is separated from the left atrium wall by epicardial fat. LCx, left circumflex artery; Thv, Thebesian valve; VV, valve of Vieussens.

The oblique vein of Marshall, a remnant of the left superior vena cava, descends along the lateral and inferior walls of the LA, between the left atrial appendage and the left PVs. It joins the cardiac vein system at the junction of the great cardiac vein and the coronary sinus (CS)**,** approximately 3 cm away from the CS ostium (Fig. **8**) [61,62]. The vein is short (2–3 cm), and its superior part can be obliterated by fibrosis. Complete fibrosis or obliteration in the form of a cord is seen in 5%–12% of cases [63]. The average diameter is 1 mm (0.4–1.8 mm), and the angle with the CS varies between 25° and 50° [64]. It is present in 85%– 95% of the population [61]. The left lateral ridge (LLR), a structure located in between the left atrial appendage and left PVs, is known to be important to AF ablation [65]. The vein or ligament of Marshall is located in the epicardial aspect of the LLR (Fig. **8**) in close proximity to the endocardial surface, at a distance of 3 mm at the superior level of the LLR in 73% of specimens. In clinical studies, electrical activity originating from the vein or ligament of Marshall can be recorded from the endocardial aspect of the LA, in or around the orifices of the left PVs. By cannulating the vein, Hwang *et al*. [49] were able to record electrical activity in patients with focal AF arising from the vein or ligament of Marshall. Postmortem human studies demonstrated multiple histological connections by myocytes muscular bundles that crossed the oblique vein to connect with the LLR, LA free wall, the CS muscle sleeves and left PVs junction [65]. Other authors have documented the electrophysiological characteristics of the vein or ligament of Marshall and its connections to the surrounding atrial structures in human patients with AF [66], and they observed that this vein or ligament may serve as a bypass tract that connects the CS to the left PVs without any LA involvement. This connection might provide a substrate for macro re-entry. Indeed, rapid electrical interaction between the left PVs and the vein or ligament of Marshall has been shown to participate in re-entry during electrically induced AF in canine models [67]. An important clinical implication of this finding is that the PV–vein or ligament of Marshall muscular connection provides an epicardial conduit between the PV and the LA through the CS muscle sleeves.

The vein or ligament of Marshall, and its adjacent epicardium, contains both autonomic nerve and muscle fibers (Fig. **8**) [65-68]. Sympathetic nerves from the middle cervical and stellate ganglia pass along the vein or ligament of Marshall to innervate the left ventricle [69]. Parasympathetic nerve fibers from the vagus nerve traverse the vein or ligament of Marshall and innervate the left atrium, left PVs, coronary sinus and posterior left atrial fat pads [70]. Cholinergic nerve fibers arising from the vein or ligament of Marshall contribute to the electrophysiological profile of surrounding LA structures [71]. Our study about the LLR also showed that the epicardial aspect of the ridge displays a higher nerve density at its superior level in relationship to the ostium of the left superior PV than at its inferior level [65] (Fig. **8**). Other authors have raised the possibility that the intrinsic cardiac nerves (superior left ganglionated plexi nerve and nerves around the vein/ligament of Marshall) can activate independently of the stellate ganglion nerve and vagal nerve activity and contribute to atrial arrhythmogenesis; in ambulatory dogs, all paroxysmal atrial tachycardia and atrial fibrillation episodes were invariably preceded by intrinsic cardiac nerves activity [72].

#### B3 Coronary Sinus

Myocardial connections between the atria ensure rapid interatrial conduction and physiologically synchronous, biatrial contraction. One important interatrial connection that has been demonstrated with anatomical and electrophysiological studies is juxtaposed posteroinferiorly to the coronary sinus (CS) [73-76]. The CS is surrounded by a myocardial sleeve**, **along 25–50 mm of its length (Fig. **8**). The CS and the oblique vein of Marshall are both remnants of the sinus venosus, and their muscle sleeve can be an extension of the RA myocardium over the CS (CS-RA muscle continuity). Other fibers of varying thicknesses arise from this sleeve and connect to the LA myocardium along the inferior mitral annulus, providing the second-largest electrical continuity between the atria (CS-LA muscle connections) [73].

The coronary sinus muscle sleeve and associated connections have been implicated in the genesis of various tachyarrhythmias, including a small minority of cases of AF [76-80]. The CS-LA muscle connections (Fig. **8**) can be the source of recurrence of AF after ablation procedures [81]. In addition, abnormal CS muscle connections with the ventricular myocardium may form atrioventricular accessory pathways and hence macro re-entrant atrial arrhythmias [80]. Most of the AF cases originating from the CS are along the CS-LA interface. Electrical disconnection of the CS-LA continuity can terminate AF that persists after pulmonary vein isolation in 30%–46% of patients [81].

#### B4 Terminal Crest

**Fig. (9):**
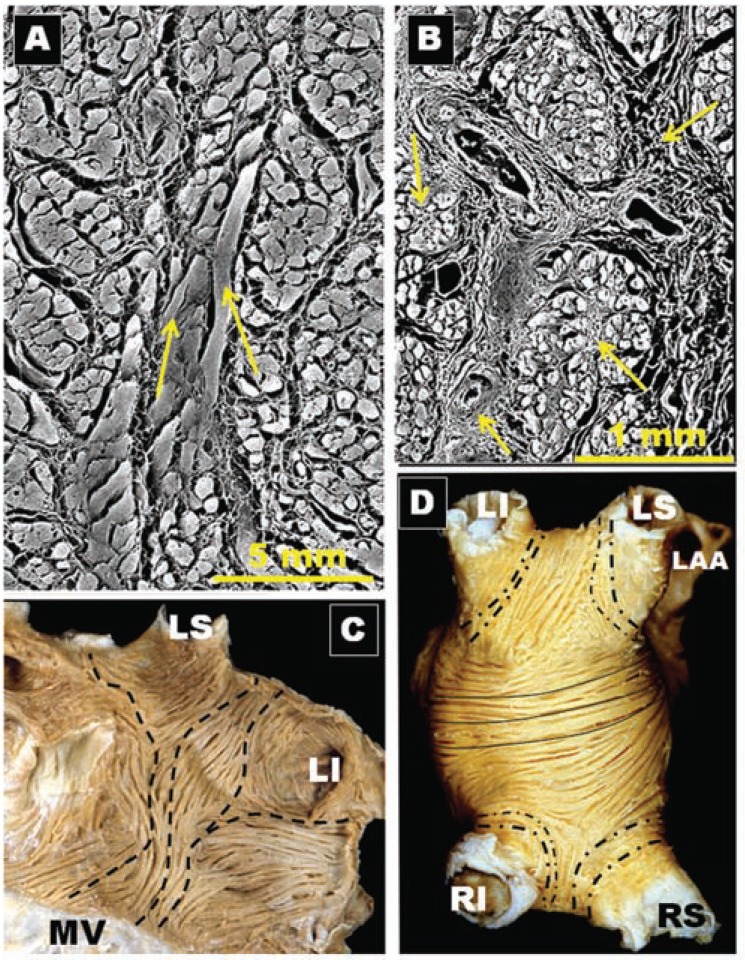
(**A**)Scanning electron micrograph of non-macerated cross section through the body of the terminal crest shows longitudinal fibers (arrows) with intermingling horizontal fibers. (**B**) Scanning electron micrograph of a cross section through the terminal crest, from a specimen of 70 years old shows a diffuse notable excess of endomysial and perimysial sheaths indicating focal interstitial reactive fibrosis (arrows). (**C**) Dissection of the subendocardium of the left atrium. The fibers ascend obliquely (broken lines) by the posterior wall of the left atrium and they have an abrupt change of direction at the level of venoatrial junctions surrounding the pulmonary veins. LI, left inferior pulmonary vein; LS, left superior pulmonary vein; MV, mitral valve. (**D**) The left atrium is everted to show the subendocardial fibers. Note that the fibers pass longitudinally over the roof of the left atrium (continuous lines). Note the abrupt change of direction of circumferential or obliquely fibers (broken lines) around the pulmonary veins. RI, right inferior pulmonary vein; RS, right superior pulmonary vein; LI, left inferior pulmonary vein; LS, left superior pulmonary vein.

Preferential conduction pathways have long been recognized in the atria, such as the terminal crest and Bachmann’s bundle [82] despite the absence of bundles of specialized conduction akin to the His-Purkinje network of the ventricles [74]. Although James and Sherf [83] attributed the faster conduction along the terminal crest and Bachmann’s bundle to the presence of specialized cells, present day thinking explains this by the anisotropic properties of atrial myocardium. The terminal crest is a significant structure in several forms of atrial tachyarrhyhmias and, occasionally, it is the target for radiofrequency catheter procedures. In common atrial flutter, the terminal crest acts as a natural barrier to conduction [84]. It may also provide a substrate that is suitable for possible re-entrant mechanisms, which could potentially promote profibrillatory remodeling [85]. Our study on the structural characteristics of the terminal crest[86] showed that the normal anatomy of the muscle fibers and connective tissue in the junctional area of the terminal crest/pectinate muscles and terminal crest/intercaval bundle favors non-uniform anisotropic properties. With advancing age, a notable diffuse excess of endomysial sheaths appear indicating focal interstitial reactive fibrosis (Fig. **9**). Other morphological studies [87] suggest that small patches of replacement fibrosis, often encountered in the terminal crest, are micro-scars of ischemic events. This phenomenon itself was not different between hearts with and hearts without a history of AF. However, the phenomenon was much more extensive in hearts from AF patients. In some AF patients, the terminal crest or Bachmann’s bundle was almost totally replaced by fibro-fatty tissue [87]. Interestingly, there is an electrophysiological study in adolescents with AF, who by age alone one would assume that the hearts are structurally normal, that exhibit spontaneous onset of AF and rapidly firing atrial foci in the PVs, terminal crest, or left atrium [88]. In post-mortem material, other researchers noted that structural changes in the atria were not associated with age, but were significantly correlated with presence of AF and its severity (patients with permanent AF had greater fibrosis extent than did patients with paroxysmal AF), and they suggest that age-related changes per se are unlikely to be the sole cause of advanced fibrosis underlying AF [89].

#### B5 Left Atrial Posterior Wall

The posterior left atrium (PLA) may be involved in the initiation and maintenance of AF. In an ovine model of acetylcholine-induced AF, Kalifa *et al*. [90] demonstrated that sites of rapid organized activity having the highest dominant frequency occurred in the PLA. At the margins of these rotors, wavebreak occurred, resulting in recording of fractionated electrograms. In an ovine model of heart failure and AF, Tanaka *et al*. [91] observed that regions of fibrosis tended to occur around the PV ostia, and that these patches could anchor re-entrant circuits and impair wave propagation, causing conduction delays and wavebreak. Markides *et al*. [77] first demonstrated that patients with AF developed a line of conduction delay on the PLA between the pulmonary veins and they suggested that this could correspond to a change in fiber orientation on this region.

The right and left atria are different from each other in their anatomical architecture. However, the right and left atrial appendages resemble each other as they are formed by pectinate muscles; but the left appendage, like the rest of the walls of the LA, has thicker myocardium than the right atrium, possibly because the left is under higher intracavitary pressure than the right [92]. Furthermore, extra-appendicular pectinate muscles are often seen in the vicinity of the ostium of the left appendage and in the inferolateral wall between the orifice of the left inferior PV and the mitral vestibule [65], and at the posterior wall of the LA containing the orifices of the four VPs and in a complex muscle architecture first described in 1920 by Papez [93]; arising from the anterosuperior septal raphe is a broad array of longitudinally to obliquely orientated myofibers that pass beneath Bachmann’s bundle to surface onto the atrial roof. The myofibers of this bundle, termed the “septopulmonary bundle” by Papez, fan out to pass in front, between and behind the insertions of the PVs, joining with the muscular sleeves of the veins (Fig. **9**). On the posterior wall, the septopulmonary bundle often becomes two diverging branches that fuse with and become indistinguishable from the circumferential myofibers coming from the lateral wall [74]. An experimental animal study by Klos *et al*. [94] showed that electrical wavefronts generated at high frequency in the PVs enter the PLA, where they encounter abrupt changes in muscle thickness and fiber direction as they move into the septopulmonary bundle. Thus, they may undergo conduction delay and wavebreak as a result of sink-to-source mismatch leading to re-entry and AF initiation. Other studies in patients with mitral regurgitation, LA enlargement and AF noted that they have more extensive regions of slow conduction in the PLA, with a constant anatomical location running vertically between PLA and the PVs [95]. They observed constant lines of conduction delay in this region leading to circuitous wavefront propagation. During persistent AF, fractionated electrograms in the PLA reside in regions demonstrating slow conduction, and the majority of these regions remain stable over time. We could confirm in our dissections that most of the hearts present an abrupt change of fibers, or mixed fibers, located in the subendocardium of the PLA towards the orifices of the PVs (Fig. **9**).

## C STRUCTURAL REMODELING THAT CONTRIBUTE TO INITIATION AND PERPETUATION OF AF

### C1 Intercellular Channels

The necessary conditions to sustain re-entry from either multiple wavelets or single high frequency rotors with fibrillatory conduction are provided by remodeling of atrial structure, myocyte sarcolemmal ion channels and intercellular communication among myocytes. Remodeling implies macroscopic structural changes in the atria such as dilatation and/or fibrosis [96,97] as well as microscopic and molecular changes in structure and function of myocytes related to altered protein synthesis of ion channel constituents. Remodeling of sarcolemmal ion channels leads to changes in atrial action potentials. Primary changes in action potentials include an acceleration of repolarization, shortening of refractory periods and a deficiency in the ability of the repolarization time course to adapt to rate changes [98]. Shortening of repolarization and refractory periods cause a decrease of the wavelength and allow small re-entrant circuits to form. Heterogeneous remodeling promotes fibrillatory conduction with inhomogeneous slow conduction and block. The concept of “electrical remodeling” has been supported by different studies [97,98], which aimed to identify the ionic current changes and action potential abnormalities associated with AF (Fig. **1**). Although the electrical remodeling of sarcolemmal ion channels during AF leads to short duration action potentials with short refractory periods, and perhaps a decrease in the sodium current, these changes alone may partially explain susceptibility to AF but not the creation of the arrhythmia. Remodeling of gap junction cell coupling may be an additional mechanism providing the substrate needed to sustain re-entry in single or multiple small circuits [99].

**Fig. (10):**
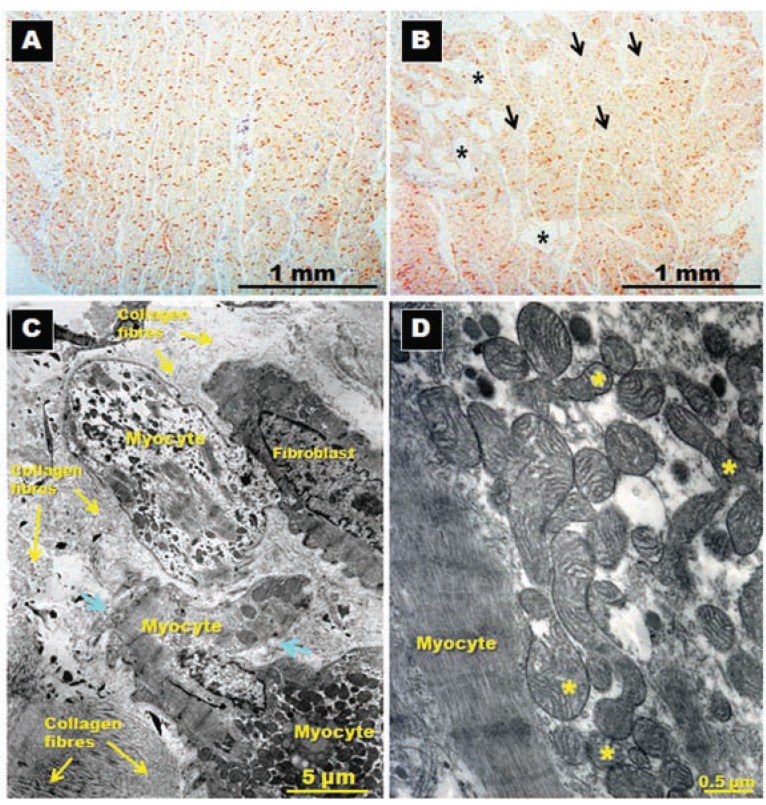
Immunohistochemical staining for N-cadherin and its distribution in posterior left atrial wall (panels **A** and **B**) sectioned in longitudinal planes to the long myocyte axis in patients in sinus rhythm (**A**) and in patients with atrial fibrillation (**B**). Note the homogenous distribution of N-cadherin in A, whereas some adipocyte cells (asterisks) and patches of myocytes with sparse and reduced levels of N-cadherin (arrows) are present in B. Panels (**C**) and ((**D**) Transmission electron micrographs in a specimen with chronic atrial fibrillation for rheumatic mitral valve disease showing in (**A**) myocytes degeneration surrounded by abundant collagen fibers, myolysis, disruption of basal membrane (arrows) and in (**D**) abnormal mitochondria with different sizes and alteration of mitochondrial cristae (asterisks).

The connexins (Cx's) and N-cadherin are a group of proteins that make up the intercellular channels between the myocytes, allowing for the transfer of small molecules and ions. Dense arrays of these channels form the gap junctions and fascia adherens, which are structures predominantly found at the intercalated disks of the cardiomyocytes and are responsible for the conduction of electrical impulses. Cx43 is the most abundant type in both atria and ventricular myocardium, whereas Cx40 is selectively found in the atria and the conduction system [7]. In a goat model of chronic AF, the distribution of Cx40 in the myocardium was discontinuous, and the heterogeneity of its distribution became more apparent with time. The overall levels of Cx40 decreased with the persistence of AF, whereas the distribution and the amount of Cx43 remained stable throughout [100]. Heterogeneity is a term that is used to describe patches in the tissue in which gap junctions or connexin labels are absent (Fig. **10**). In 31 patients with chronic AF undergoing a Maze procedure, Kostin *et al*. [101] observed a reduction of Cx43. A heterogeneous distribution of Cx40 and N-cadherin was found, with variable amounts of Cx40 in different right atrium tissues or in spatially adjacent regions of atrial myocardium. The gap junctions in the atrial myocardium of the caval veins and PVs are composed of Cx40 and Cx43 [57,102]. Levels of Cx43 are similar to those found in the atria, whereas levels of Cx40 within the PVs are lower than those found in the atria [102]. This heterogeneous distribution may contribute to slow conduction in the veins leading to re-entry. Studies in humans confuse the picture still further, with reports linking AF variously to increased Cx40, decreased Cx40, increased Cx43, decreased Cx43 or to no change in the level of either connexin. In addition, some studies report lateralization of gap junction distribution, whereas others claim increased heterogeneity [98]. As Severs *et al*. [103] discussed in a recent review paper, it is important to emphasize that the published studies on animal models use different species at different ages, with different experimental protocols for induction of AF, and those on the human involve different clinical sub-sets of patients with different associated pathological factors.

The observed ionic changes and remodeling of gap junction cell coupling provide little evidence for the initiation of the arrhythmia. The concept of a “second factor” beyond the electrophysiological alterations associated with AF has involved the role of atrial anatomical changes to provide the susceptible substrate for the arrhythmia. These changes are identified at the level of myocytes and extracellular matrix, and it is not clear whether they precede or follow the development of the arrhythmia.

### C2 Myocite Degeneration-Fibrosis

The concept of fibrosis and myocyte degeneration in AF has been studied in humans with operated mitral valve disease [104]. Thiedemann and Ferrans described that myocytes, located within fibrotic areas all around the LA, tended to be isolated from adjacent cells and exhibited myolysis of varying severity. These changes consisted of proliferation of Z-band material and cytoskeletal filaments, myofibrillar loss, variation in size and number of mitochondria, occurrence of abnormal mitochondria, dissociation of intercellular junctions, and accumulation of lysosomal degradation products. The severity of degeneration was greater in patients with mitral regurgitation than in patients with pure mitral stenosis. We have observed similar results in left atrial biopsies of patients with mitral stenosis before mitral surgery [105] (Fig. **10**). However, these changes may be due to the underlying mitral valve disease, of which AF is a common sequence, rather than due to the arrhythmia itself.

**Fig. (11):**
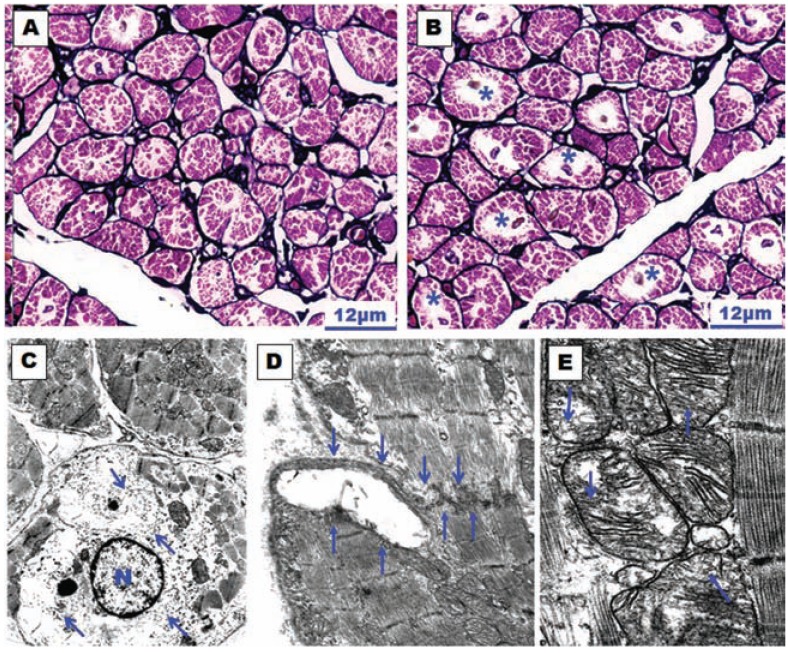
(**A**) and (**B**)Histological sections of the right atrial appendage with silver-hematoxylin staining. (**A**) Dog control group in which little myolysis is observed. (**B**) Dog stimulated group (atrially paced at 400/min for 3 days) in which the myolysis is much more evident around the nucleus of the myocyte (asterisks). (**C**), (**D**) and (**E**) Transmission electron micrographs of the atrial wall observed in a dog subjected to atrial pacing for 3 days. In (**C**) note the perinuclear myolysis, perinuclear accumulation of glycogen grains (arrows) and nuclear chromatin dispersion. N, nucleus of the myocyte. Magnification x8000. In (**D**) disruption of an intercalated disc (between arrows). Magnification x10500. In (**E**) the mitochondria have different sizes and structural alterations with marked dilatation and disruption of mitochondrial cristae (arrows). Magnification x17500.

In an canine model of chronic AF (atrially paced at a rate of 400/min for 6 weeks), which was developed and studied by Morillo *et al*. [106], atrial structural changes and electrophysiological abnormalities were associated with the sustained arrhythmia. The structural changes were: atrial dilatation, myocytolytic changes, increased number and size of mitochondria, enlarged nuclei, and abnormalities in both the sarcoplasmic and rough endoplasmic reticula. However, no significant changes were seen in the extracellular matrix (i.e. interstitial fibrosis). In a goat model of chronic AF (for 9 to 23 weeks), the above findings were confirmed, and myolysis, glycogen accumulation and chromatin changes were also demonstrated [107]. The more homogenous distribution of heterochromatin in the cell nucleus of the abnormal myocytes resembled embryonic levels of development, leading to the notion that AF is associated with dedifferentiation of myocytes rather than degeneration. This dedifferentiation of myolytic myocytes has also been described in a study of human atrial myocardium [108]. The findings of interstitial fibrosis appear to be only evident in animal models of chronic AF^109^. In fact in our experimental model (continuous atrially paced at 400/min for 3 days in dogs), we observed structural alterations similar to those models of chronic AF. However, in contrast to the previous studies, we did not observe atrial dilatation, myocyte hypertrophy or interstitial fibrosis [110] (Fig. **11**).

Atrial fibrillation may lead to progressive dilatation of the atria, which in turn may promote stabilization of the arrhythmia. However, the mechanisms of this vicious cycle are poorly understood. Based on studies of chronic AF in a goat model, Schotten *et al*. [111] proposed that dilatation of the atria is an early mechanism in the maintenance of AF caused by a loss of contractility and a decrease in compliance of the atria during fibrillation. These authors also demonstrated that both the compliance and the size of the atria recovered to normal after cardioversion. The chronic dilatation of the atria induces the activation of many signaling pathways leading to hypertrophy, proliferation of fibroblasts and fibrosis [112]. Atrial dilatation is associated with an increase in the heterogeneity of the impulse propagation and with a decreased impulse rate, which can promote re-entry [113]. Kalifa *et al*. [114] showed that when there was dilatation and stretching of the atria, the sources of rapid activation that sustained AF were located at the junction of the pulmonary veins and the posterior wall of the LA.

**Fig. (12):**
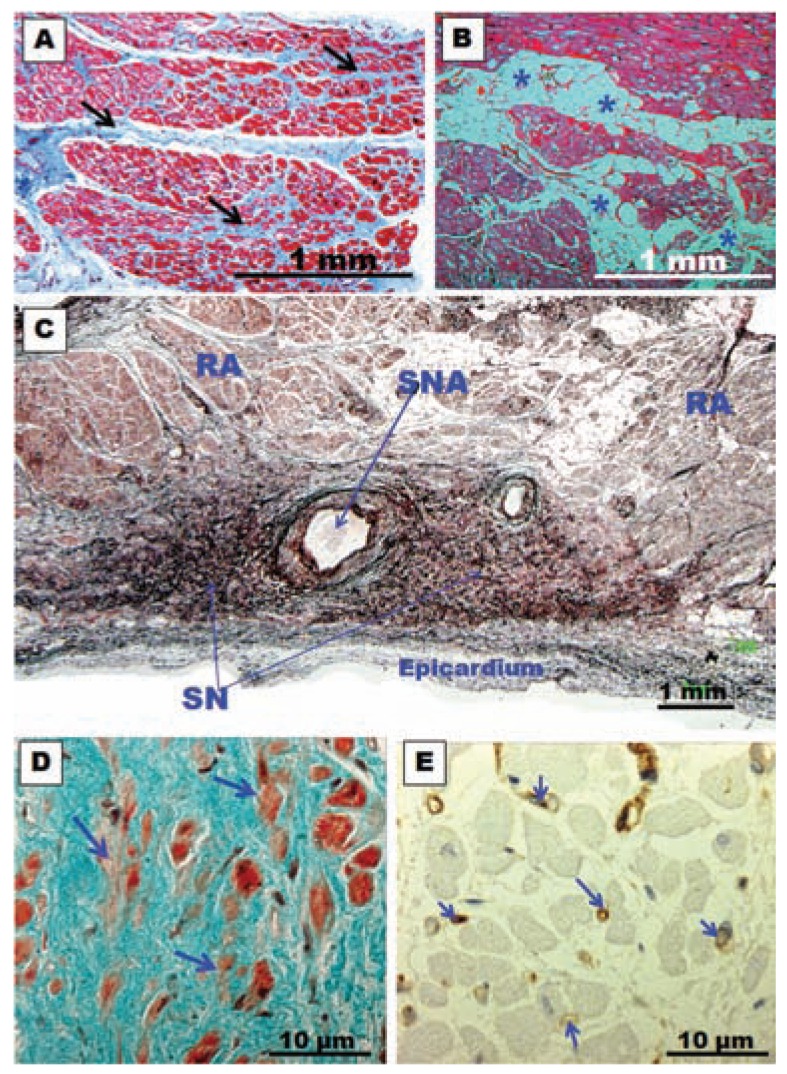
(**A**) and (**B**)Histological cross-section biopsy samples from the posterior left atrial wall in specimens with chronic atrial fibrillation for rheumatic mitral valve disease. Note in (**A**) the abundant connective tissue between the myocytes (arrows) and interstitial fibrosis. In (**B**) note accumulation of fat cells (asterisks) between myocytes. (**C**) Histological section of the sinus node with methenamine silver staining of a specimen with chronic atrial fibrillation. Note the intense accumulation of connective tissue between nodal cells. SN, sinus node; SNA, sinus node artery; RA, right atrium. (**D**) Sinus node. Trichrome stain. Note the abundant connective tissue and fewer larger myocardial nodal cells (arrows) in a patient with long-term chronic atrial fibrillation. (**E**) Immunohistochemical staining for CD31 (vessel walls stained in brown) of the sinus node in a patient with long-term chronic atrial fibrillation. Note fewer and thinner capillaries in the sinus node (arrows).

Interstitial fibrosis creates conduction delay causing the electrical impulse to propagate through alternative pathways and eventually impinge on tissue that has already recovered excitability, causing reactivation and further increase in the number of re-entrant circuits [1,6]. Histological data from biopsies [105] (Fig. **12**) and autopsy specimens have established a good relationship between AF and the presence of fibrosis [101], showing an increased degree of fibrosis in the atria of patients with AF compared with those which remain in sinus rhythm [115]. The abundance of collagen provides an appropriate substrate by creating areas of different conduction properties within the atria. Collagen synthesis and degradation are influenced by the action of matrix metalloproteinases, and also by pro-fibrotic signals stimulating the proliferation of fibroblasts. Other factors include: the presence of altered expression of matrix proteins other than collagen such as fibronectin 1 and fibrillin 1, deposition of proteoglycans and other extracellular matrix components. At a molecular level, it is not well known which signaling pathways mediate the development of fibrosis. Nevertheless, it has been demonstrated that the atria are more susceptible to the development of fibrosis than the ventricles. 

Currently, three pathways related to each other have been described as the most important mechanisms in pathological fibrosis of the atria: 1) The role of the renin-angiotensin system has been previously demonstrated in the cardiovascular system [116], and it is likely that the establishment of fibrosis in the atrial tissue during AF could be related to changes in concentration of the subtype 1 of the angiotensin II receptor (AT1) in the atrial myocardium [117]. 2) The role of oxidative stress in the pathogenesis and perpetuation of AF. Several pathophysiological changes possibly associated with increased oxidative stress in AF have been proposed. These include changes in gene transcriptional profiles and mitochondrial DNA, increased activity of enzymes such as NAD(P)H oxidase and xanthine oxidase, and inflammatory processes. Preliminary studies using dietary antioxidants such as vitamin C have shown promising results [118]. 3) The association of transforming growth factor β1(TGF-β1) with production of extracellular matrix proteins and tissue fibrosis has also been described [119]. In transgenic mice with increased levels of myocardial TGF-β1, Nakajima *et al*. [120] demonstrated increased fibrosis in the atria, which was not paired with anticipated fibrosis of the ventricles. Despite the observed hypertrophy of ventricular myocytes, no fibrosis was present in the ventricular myocardium, which indicates the differential effect of TGF-β1 on atrial over ventricular myocardium, leading to selective atrial structural remodeling.

### C3 Sinus Node Function

There is a clinical association between abnormalities of sinus node (SN) function and AF. At one time, this observation and the characteristic slow conduction within the SN, led investigators to speculate that the SN might be involved in maintaining the arrhythmia. According to Davies and Pomerance [121] there is a difference in the SN between long-term AF and short term AF. For these authors, the SN is normal in short-term AF, whereas there is reduced percentage of specialized myocytes in long-term AF. According to the authors, it is possible that the fibrotic changes in the node and atria result from the arrhythmia and consequent disordered function of the chambers. Other authors showed that the nodal fibers percentage is similar in the sinus rhythm group and in the AF patient group [122]. The clinical data available at present suggest that the SN is probably passive during AF, with atrial impulses invading the SN at a rate much faster than its intrinsic frequency. The association between SN dysfunction and AF is probably due to diseases that affect both the SN and atria simultaneously, rather than participation of SN pathology per se in AF [1]. We studied the SN microscopic features of patients with long-term permanent AF [123,124]. Our findings can be synthesized into four main features: (1) progressive tissue fibrosis, (2) progressive myocardial cell loss (including P cells), (3) myocardial cell degeneration (including P cells) (4) significant reduction in the amount of capillaries in the SN (Fig. **12**). These SN morphological abnormalities in patients with chronic AF must inevitably lead to an impairment of its function. Thus normal sinus rhythm is less likely to be restored in patients with long-standing AF, regardless of the atrial lesion pattern used to interrupt re-entry or the energy source used to create these lesions because their SN is morphologically and functionally abnormal. Conversely, surgical treatment of AF during its early stages, with minimal SN damage, is more likely to be successful.

### C4 Inflammation and Atrial Myocarditis

The concept that inflammation contributes to at least some types of AF is supported by the frequent occurrence of AF after cardiac surgery (25% to 40%) [125], the genetic studies linking inflammation to AF [126] and the association of AF with pericarditis [127]. The first study to support the role of inflammation in AF pathogenesis was reported in 1997, when Frustaci *et al*. [128] observed inflammatory infiltrates, myocyte necrosis, and fibrosis in atrial biopsies of patients with lone AF refractory to antiarrhythmic drug therapy. 

C-reactive protein (CRP) is a component of the innate immune system, an acute-phase protein produced in the liver as a response to interleukins (ILs) 1 and 6. Interleukin 6 (IL-6) is a proinflammatory cytokine that is responsible for the synthesis of acute-phase proteins (such as CRP) while it also exhibits cytoprotective properties. Higher plasma IL-6 levels were identified in patients with AF compared with controls in a study by Conway *et al*. [129]. Other studies such as the one published by Aviles *et al*. [130] showed that baseline CRP levels were significantly and independently associated with the development of future AF. Chung *et al*. [131] demonstrated that CRP was also significantly higher in the arrhythmia group. Moreover, patients belonging to the persistent AF group had higher CRP levels than the paroxysmal AF and the control groups, indicating the possible relationship between CRP levels and chronicity of AF. A possible mechanism by which CRP can induce AF involves the disarray of normal cell membrane structure in conditions that cause energy depletion and apoptosis, such as ischemia and oxidative stress [132]. These changes progressively lead to loss of atrial muscle mass and interstitial fibrosis, which are known determinants of AF, associating the inflammatory response with structural remodeling. Whether initiation of AF activates direct inflammatory effects or whether the presence of a preexisting systemic inflammatory state promotes further persistence of AF remains unclear. This low-level inflammatory response may thus be part of the structural remodeling process associated with increased persistence of AF [6,133]. Alternatively, the presence of an elevated baseline level of systemic inflammation may predispose patients with triggering atrial foci to the development of persistent AF [130]. 

## CONCLUSIONS

Atrial fibrillation is a complex disease most probably due to multiple aetiopathogenic mechanisms. In spite of an incomplete understanding of the anatomo-functional basis for AF, current evidence demonstrates that this arrhythmia usually requires a trigger for initiation and a vulnerable electrophysiological and/or anatomical substrate for maintenance. It is still unclear whether the trigger mechanisms include focal enhanced automaticity, triggered activity or micro re-entry from myocardial tissue. Initiation of AF can be favored by both parasympathetic and sympathetic stimulation, which seem to play a role also in maintaining AF.

Different atrial regions contribute to the fibrillatory process and to the maintenance of AF, emphasizing the role of structural discontinuities and heterogeneous fiber orientation transmurally along the myocardial bundles, favoring anatomical re-entry or anchoring rotors. Despite electrical remodeling being a reversible process, the structural remodeling, with features of interstitial fibrosis and cellular degeneration provides a susceptible substrate that promotes re-entrant circuits and AF. Finally, evolving clinical evidence demonstrates that inflammation is associated with new-onset and recurrent AF through a mechanism that possibly involves cellular degeneration, apoptosis, and subsequent atrial fibrosis.

## References

[1] Nattel S (2002). New ideas about atrial fibrillation 50 years on. Nature.

[2] Haïssaguerre M, Jaïs P, Shah DC (1998). Spontaneous initiation of atrial fibrillation by ectopic beats originating in the pulmonary veins. N Engl J Med.

[3] Hwang C, Wu TJ, Doshi RN, Peter CT, Chen PS (2000). Vein of Marshall cannulation for the analysis of electrical activity in patients with focal atrial fibrillation. Circulation.

[4] Allessie MA, Bonke FI, Schopman FJ (1977). Circus movement in rabbit atrial muscle as a mechanism of tachycardia. III. The leading circle concept: a new model of circus movement in cardiac tissue without the involvement of an anatomical obstacle. Circ Res.

[5] Wijffels MC, Kirchhof CJ, Dorland R (1995). Atrial fibrillation begets atrial fibrillation. A study in awake chronically instrumented goats. Circulation.

[6] Allessie M, Ausma J, Schotten U (2002). Electrical, contractile and structural remodeling during atrial fibrillation. Cardiovasc Res.

[7] Kourliouros A, Savelieva I, Kiotsekoglou A (2009). Current concepts in the pathogenesis of atrial fibrillation. Am Heart J.

[8] Scharf C, Sneider M, Case I (2003). Anatomy of the pulmonary veins in patients with atrial fibrillation and effects of segmental ostial ablation analyzed by computed tomography. J Cardiovasc Electrophysiol.

[9] Cronin P, Sneider MB, Kazerooni EA (2004). MDCT of the Left Atrium and Pulmonary Veins in Planning Radiofrequency Ablation for Atrial Fibrillation: A How-To Guide. AJR Am J Roentgenol.

[10] Tsao HM, WU MH, Yu WC (2001). Role of right middle pulmonary vein in patients with paroxysmal atrial fibrillation. J Cardiovasc Electrophysiol.

[11] Marom EM, Herndon JE, Kim YH (2004). Variations in pulmonary venous drainage to the left atrium: implications for radiofrequency ablation. Radiology.

[12] Ho SY, Cabrera JA, Sánchez-Quintana D, Chen SA, Haïssaguerre M, Zipes DP (2004). Anatomy of the pulmonary vein-atrium junction. Thoracic vein arrhythmias. Mechanisms and treatment.

[13] Oral H, Pappone C, Chugh A (2006). Circumferential pulmonary-vein ablation for chronic atrial fibrillation. N Engl J Med.

[14] Fisher JD, Spinelli MA, Mookherjee D, Krumerman AK, Palma EC (2006). Atrial fibrillation ablation: reaching the mainstream. Pacing Clin Electrophysiol.

[15] Lim KT, Matsuo S, O'Neill MD (2007). Catheter ablation of persistent and permanent atrial fibrillation: Bordeaux experience. Expert Rev Cardiovasc Ther.

[16] O'Neill MD, Jaïs P, Takahashi Y (2006). The stepwise ablation approach for chronic atrial fibrillation evidence for a cumulative effect. J Interv Card Electrophysiol.

[17] Nathan H, Eliakim M (1966). The junction between the left atrium and the pulmonary veins. An anatomic study of human Hearts. Circulation.

[18] Ho SY, Sánchez-Quintana D, Cabrera JA (1999). Anatomy of the left atrium: Implications for radiofrequency ablation of atrial fibrillation. J Cardiovasc Electrophysiol.

[19] Ho SY, Cabrera JA, Tran VH (2001). Architecture of the pulmonary veins: Relevance to radiofrequency ablation. Heart.

[20] Cabrera JA, Ho SY, Climent V (2009). Morphological evidence of muscular connections between contiguous pulmonary venous orifices: relevance of the interpulmonary isthmus for catheter ablation in atrial fibrillation. Heart Rhythm.

[21] Valles E, Fan R, Roux JF (2008). Localization of atrial fibrillation triggers in patients undergoing pulmonary vein isolation: importance of the carina region. J Am Coll Cardiol.

[22] Rajappan K, Kistler P, Earley M (2008). Acute and chronic pulmonary vein reconnection after atrial fibrillation ablation: a prospective characterization of anatomical sites. Pacing Clin Electrophysiol.

[23] Hassink RJ, Aretz HT, Ruskin J, Keane D (2003). Morphology of atrial myocardium in human pulmonary veins: a postmortem analysis in patients with and without atrial fibrillation. J Am Coll Cardiol.

[24] Saito T, Waki K, Becker A (2000). Left atrial myocardial extension onto pulmonary veins in humans: Anatomic observations relevant for atrial arrhythmias. J Cardiovasc Electrophysiol.

[25] Tagawa M, Higuchi K, Chinushi M (2001). Myocardium extending from the left atrium onto the pulmonary veins: A comparison between subjects with and without atrial fibrillation. Pacing Clin Electrophysiol.

[26] Kholová I, Kautzner J (2003). Anatomic characteristics of extensions of atrial myocardium into the pulmonary veins in subjects with and without atrial fibrillation. Pacing Clin Electrophysiol.

[27] Steiner I, Hájková P, Kvasnicka J, Kholová I (2006). Myocardial sleeves of pulmonary veins and atrial fibrillation: a postmortem histopathological study of 100 subjects. Virchows Arch.

[28] Spach MS, Boineau JP (1997). Microfibrosis produces electrical load variations due to loss of side-to-side cell connections: a major mechanism of structural heart disease arrhythmias. Pacing Clin Electrophysiol.

[29] Pérez-Lugones A, McMahon JT, Ratliff NB (2003). Evidence of specialized conduction cells in human pulmonary veins of patients with atrial fibrillation. J Cardiovasc Electrophysiol.

[30] Chou CC, Nihei M, Zhou S (2005). Intracellular calcium dynamics and anisotropic reentry in isolated canine pulmonary veins and left atrium. Circulation.

[31] Chou C-C, Nguyen BL, Tan AY (2008). Intracellular calcium dynamics and acetylcholine-induced triggered activity in the pulmonary veins of dogs with pacing-induced heart failure. Heart Rhythm.

[32] Monckeberg JG (1910). Beitrage zur normalen und pathologischen anatomie des herzens. Verh Dtsch Pathol Ges.

[33] Aschoff L (1910). Referat uber die herzstorungen in ihren beziehungen zu den spezifischen muskelsystem des herzens. Verh Dtsch Pathol Ges.

[34] Sánchez-Quintana D, Ho SY (2003). Anatomy of cardiac nodes and atrioventricular specialized conduction system. Rev Esp Cardiol.

[35] Morel E, Meyronet D, Thivolet-Bejuy F, Chevalier P (2008). Identification and distribution of interstitial Cajal cells in human pulmonary veins. Heart Rhythm.

[36] Gherghiceanu M, Hinescu ME, Andrei F (2008). Interstitial Cajal-like cells (ICLC) in myocardial sleeves of human pulmonary veins. J Cell Mol Med.

[37] Levin MD, Lu MM, Petrenko NB (2009). Melanocyte-like cells in the heart and pulmonary veins contribute to atrial arrhythmia triggers. J Clin Invest.

[38] Zimmermann M, Kalusche D (2001). Fluctuation in autonomic tone is a major determinant of sustained atrial arrhythmias in patients with focal ectopy originating from the pulmonary veins. J Cardiovasc Electrophysiol.

[39] Lu Z, Scherlag BJ, Lin J (2009). Autonomic mechanism for initiation of rapid firing from atria and pulmonary veins: evidence by ablation of ganglionated plexi. Cardiovasc Res.

[40] Vaitkevicius R, Saburkina I, Rysevaite K (2009). Nerve supply of the human pulmonary veins: an anatomical study. Heart Rhythm.

[41] Ogawa M, Zhou S, Tan AY (2007). Left stellate ganglion and vagal nerve activity and cardiac arrhythmias in ambulatory dogs with pacing-induced congestive heart failure. J Am Coll Cardiol.

[42] Nguyen BL, Fishbein MC, Chen LS (2009). Histopathological substrate for chronic atrial fibrillation in humans. Heart Rhythm.

[43] Oh S, Zhang Y, Bibevski S (2006). Vagal denervation and atrial fibrillation inducibility: epicardial fat pad ablation does not have long-term effects. Heart Rhythm.

[44] Edgerton JR, Jackman WM, Mack MJ (2007). Minimally invasive pulmonary vein isolation and partial autonomic denervation for surgical treatment of atrial fibrillation. J Interv Card Electrophysiol.

[45] Cabrera JA, Pizarro G, Sánchez-Quintana D (2010). Transmural ablation of all the pulmonary veins: is it the Holy Grail for cure of atrial fibrillation?. Eur Heart J.

[46] Tsai CF, Tai CT, Hsieh MH (2000). Initiation of atrial fibrillation by ectopic beats originating from the superior vena cava: electrophysiological characteristics and results of radiofrequency ablation. Circulation.

[47] Lin WS, Tai CT, Hsieh MH (2003). Catheter ablation of paroxysmal atrial fibrillation initiated by non-pulmonary vein ectopy. Circulation.

[48] Johnson N, Danilo P, Wit AL, Rosen MR (1986). Characteristics of initiation and termination of catecholamine-induced triggered activity in atrial fibers of the coronary sinus. Circulation.

[49] Hwang C, Wu TL, Doshi RN (2000). Vein of Marshall cannulation for the analysis of electrical activity in patients with focal atrial fibrillation. Circulation.

[50] Katritsis D, Ioannidis JPA, Anagnostopoulos CE (2001). Identification and catheter ablation of extracardiac and intracardiac components of ligament of Marshall tissue for treatment of paroxysmal atrial fibrillation. J Cardiovasc Electrophysiol.

[51] Kurotobi T, Iwakura K, Inoue K (2010). Multiple arrhythmogenic foci associated with the development of perpetuation of atrial fibrillation. Circ Arrhythm Electrophysiol.

[52] Di Biase L, Burkhardt JD, Mohanty P (2010). Left Atrial Appendage: An Underrecognized Trigger Site of Atrial Fibrillation. Circulation.

[53] Spach MS, Barr RC, Jewett PH (1972). Spread of excitation from the atrium into thoracic veins in human beings and dogs. Am J Cardiol.

[54] Chen YJ, Chen YC, Yeh HI (2002). Electrophysiology and arrhythmogenic activity of single cardiomyocytes from canine superior vena cava. Circulation.

[55] Kholová I, Kautzner J (2004). Morphology of atrial myocardial extensions into human caval veins: a postmortem study in patients with and without atrial fibrillation. Circulation.

[56] Huang BH, Wu MH, Tsao HM (2005). Morphology of the thoracic veins and left atrium in paroxysmal atrial fibrillation initiated by superior caval vein ectopy. J Cardiovasc Electrophysiol.

[57] Yeh HI, Lai YJ, Lee SH, Lee YN (2001). Heterogeneity of myocardial sleeve morphology and gap junctions in canine superior vena cava. Circulation.

[58] Aanhaanen WT, Mommersteeg MT, Norden J (2010). Developmental origin, growth, and three-dimensional architecture of the atrioventricular conduction axis of the mouse heart. Circ Res.

[59] Matsuyama TA, Inoue S, Kobayashi Y (2004). Anatomical diversity and age-related histological changes in the human right atrial posterolateral wall. Europace.

[60] Sánchez-Quintana D, Cabrera JA, Farré J (2005). Sinus node revisited in the era of electroanatomical mapping and catheter ablation. Heart.

[61] de Oliveira IM, Scanavacca MI, Correia AT (2007). Anatomic relations of the Marshall vein: importance for catheterization of the coronary sinus in ablation procedures. Europace.

[62] Kim DT, Lai AC, Hwang C (2000). The ligament of Marshall: a structural analysis in human hearts with implications for atrial arrhythmias. J Am Coll Cardiol.

[63] von Lüdinghausen M (2003). The venous drainage of the human myocardium. Adv Anat Embryol Cell Biol.

[64] Saremi F, Muresian H, Sánchez-Quintana D (2012). Coronary veins: comprehensive CT-anatomic classification and review of variants and clinical implications. Radiographics.

[65] Cabrera JA, Ho SY, Climent V, Sánchez-Quintana D (2008). The architecture of the left lateral atrial wall: a particular anatomic region with implications for ablation of atrial fibrillation. Eur Heart J.

[66] Han S, Joung B, Scanavacca M (2010). Electrophysiological characteristics of the Marshall bundle in humans. Heart Rhythm.

[67] Tan AY, Chou CC, Zhou S (2006). Electrical connections between left superior pulmonary vein, left atrium, and ligament of Marshall: implications for mechanisms of atrial fibrillation. Am J Physiol Heart Circ Physiol.

[68] Doshi RN, Wu TJ, Yashima M (1999). Relation between ligament of Marshall and adrenergic atrial tachyarrhythmia. Circulation.

[69] Armour JA, Richer LP, Page P (2005). Origin and pharmacological response of atrial tachyarrhythmias induced by activation of mediastinal nerves in canines. Auton Neurosci.

[70] Ulphani JS, Arora R, Cain JH (2007). The ligament of Marshall as a parasympathetic conduit. Am J Physiol Heart Circ Physiol.

[71] Lin J, Scherlag BJ, Lu Z (2008). Inducibility of atrial and ventricular arrhythmias along the ligament of marshall: role of autonomic factors. J Cardiovasc Electrophysiol.

[72] Choi EK, Shen MJ, Han S (2010). Intrinsic cardiac nerve activity and paroxysmal atrial tachyarrhythmia in ambulatory dogs. Circulation.

[73] Chauvin M, Shah DC, Haïssaguerre M  (2000). The anatomic basis of connections between the coronary sinus musculature and the left atrium in humans. Circulation.

[74] Ho SY, Sánchez-Quintana D (2009). The importance of atrial structure and fibers. Clin Anat.

[75] Antz M, Otomo K, Arruda A (1998). Electrical conduction between the right atrium and the left atrium via the musculature of the coronary sinus. Circulation.

[76] Oral H, Ozaydin M, Chugh A (2003). Role of the coronary sinus in maintenance of atrial fibrillation. J Cardiovasc Electrophysiol.

[77] Markides V, Schilling RJ, Ho SY (2003). Characterization of left atrial activation in the intact human heart. Circulation.

[78] Kasai A, Anselme F, Saoudi N (2001). Myocardial connections between left atrial myocardium and coronary sinus musculature in man. J Cardiovasc Electrophysiol.

[79] Katritsis DG (2004). The coronary sinus: passive bystander or source of arrhythmia?. Heart Rhythm.

[80] Sun Y, Arruda M, Otomo K (2002). Coronary sinus-ventricular accessory connections producing posteroseptal and left posterior accessory pathways: incidence and electrophysiological identification. Circulation.

[81] Haïssaguerre M, Hocini M, Takahashi Y (2007). Impact of catheter ablation of the coronary sinus on paroxysmal or persistent atrial fibrillation. J Cardiovasc Electrophysiol.

[82] Boineau JP, Canavan TE, Schuessler RB (1988). Demonstration of a widely distributed atrial pacemaker complex in the human heart. Circulation.

[83] Sherf L, James TN (1979). Fine structure of cells and their histologic organization within internodal pathways of the heart: clinical and electrocardiographic implications. Am J Cardiol.

[84] Olgin JE, Kalman JM, Fitzpatrick A (1995). Role of right atrial endocardial structures as barriers to conduction during human type I atrial flutter: activation and entrainment mapping guided by intracardiac echocardiography. Circulation.

[85] Saoudi N, Cosio F, Waldo A (2001). Classification of atrial flutter and regular atrial tachycardia according to electrophysiologic mechanism and anatomic bases: a statement from a joint expert group from the Working Group of Arrhythmias of the European Society of Cardiology and the North American Society of Pacing and Electrophysiology. J Cardiovasc Electrophysiol.

[86] Sánchez-Quintana D, Anderson RH, Cabrera JA (2002). The terminal crest: morphological features relevant to electrophysiology. Heart.

[87] Becker AE (2004). How structurally normal are human atria in patients with atrial fibrillation?. Heart Rhythm.

[88] Nanthakumar K, Lau YR, Plumb VJ (2004). Electrophysiological findings in adolescents with atrial fibrillation who have structurally normal hearts. Circulation.

[89] Platonov PG, Mitrofanova LB, Orshanskaya V, Ho SY (2011). Structural abnormalities in atrial walls are associated with presence and persistency of atrial fibrillation but not with age. J Am Coll Cardiol.

[90] Kalifa J, Tanaka K, Zaitsev AV (2006). Mechanisms of wave fractionation at boundaries of high-frequency excitation in the posterior left atrium of the isolated sheep heart during atrial fibrillation. Circulation.

[91] Tanaka K, Zlochiver S, Vikstrom KL (2007). Spatial distribution of fibrosis governs fibrillation wave dynamics in the posterior left atrium during heart failure. Circ Res.

[92] Jalife J (2009). ¿Por qué es la aurícula izquierda tan importante en el mecanismo de la fibrilación auricular crónica?. Revista Iberoamericana de Arritmologa - ria.

[93] Papez J (1920). Heart musculature of the atria. Am J Anat.

[94] Klos M, Calvo D, Yamazaki M (2008). Atrial septopulmonary bundle of the posterior left atrium provides a substrate for atrial fibrillation initiation in a model of vagally mediated pulmonary vein tachycardia of the structurally normal heart. Circ Arrhythm Electrophysiol.

[95] Roberts-Thomson KC, Stevenson I, Kistler PM (2009). The role of chronic atrial stretch and atrial fibrillation on posterior left atrial wall conduction. Heart Rhythm.

[96] Everett TH, Olgin JE (2007). Atrial fibrosis and the mechanisms of atrial fibrillation. Heart Rhythm.

[97] Xu J, Cui G, Esmailian F (2004). Atrial extracellular matrix remodeling and the maintenance of atrial fibrillation. Circulation.

[98] Nattel S, Maguy A, Le Bouter S, Yeh YH (2007). Arrhythmogenic ion-channel remodeling in the heart: heart failure, myocardial infarction, and atrial fibrillation. Physiol Rev.

[99] Duffy HS, Wit AL (2008). Is there a role for remodeled connexins in AF?. No simple answers. J Mol Cell Cardiol.

[100] van der Velden HM, Ausma J, Rook MB (2000). Gap junctional remodeling in relation to stabilization of atrial fibrillation in the goat. Cardiovasc Res.

[101] Kostin S, Klein G, Szalay Z (2002). Structural correlate of atrial fibrillation in human patients. Cardiovasc Res.

[102] Verheule S, Wilson EE, Arora R, Engle SK, Scott LR, Olgin JE (2002). Tissue structure and connexin expression of canine pulmonary veins. Cardiovasc Res.

[103] Severs NJ, Bruce AF, Dupont E, Rothery S (2008). Remodelling of gap junctions and connexin expression in diseased myocardium. Cardiovasc Res.

[104] Thiedemann KU, Ferrans VJ (1977). Left atrial ultrastructure in mitral valvular disease. Am J Pathol.

[105] Climent V, Hurlé A, Ho SY (2004). Early morphologic changes following microwave endocardial ablation for treatment of chronic atrial fibrillation during mitral valve surgery. J Cardiovasc Electrophysiol.

[106] Morillo CA, Klein GJ, Jones DL (1995). Chronic rapid atrial pacing.
Structural, functional, and electrophysiological characteristics of a
new model of sustained atrial fibrillation. Circulation.

[107] Ausma J, Wijffels M, Thone F (1997). Structural changes of atrial myocardium due to sustained atrial fibrillation in the goat. Circulation.

[108] Rucker-Martin C, Pecker F, Godreau D (2002). Dedifferentiation of atrial myocytes during atrial fibrillation: role of fibroblast proliferation in vitro. Cardiovasc Res.

[109] Ausma J, Wijffels M, van Eys G (1997). Dedifferentiation of atrial cardiomyocytes as a result of chronic atrial fibrillation. Am J Pathol.

[110] Cabrera JA, Climent V, Murillo M (2010). Alteraciones estructurales precoces asociadas al remodelado eléctrico de la fibrilación auricular en un modelo canino de estimulación auricular sostenida a altas frecuencias. Efecto protector del Irbesartn. Revista Iberoamericana de Arritmologa - ria.

[111] Schotten U, de Haan S, Neuberger HR (2004). Loss of atrial contractility is primary cause of atrial dilatation during first days of atrial fibrillation. Am J Physiol Heart Circ Physiol.

[112] Eckstein J, Verheule S, de Groot NM (2008). Mechanisms of perpetuation of atrial fibrillation in chronically dilated atria. Prog Biophys Mol Biol.

[113] Eijsbouts SC, Majidi M, van Zandvoort M, Allessie MA (2003). Effects of acute atrial dilation on heterogeneity in conduction in the isolated rabbit heart. J Cardiovasc Electrophysiol.

[114] Kalifa J, Jalife J, Zaitsev AV (2003). Intra-atrial pressure increases rate and organization of waves emanating from the superior pulmonary veins during atrial fibrillation. Circulation.

[115] Boldt A, Wetzel U, Lauschke J (2004). Fibrosis in left atrial tissue of patients with atrial fibrillation with and without underlying mitral valve disease. Heart.

[116] Weber KT, Brilla CG (1991). Pathological hypertrophy and cardiac interstitium. Fibrosis and renin-angiotensin-aldosterone system. Circulation.

[117] Tsutsumi Y, Matsubara H, Ohkubo N (1998). Angiotensin II type 2 receptor is upregulated in human heart with interstitial fibrosis, and cardiac fibroblasts are the major cell type for its expression. Circ Res.

[118] Korantzopoulos P, Kolettis TM, Galaris D, Goudevenos JA (2007). The role of oxidative stress in the pathogenesis and perpetuation of atrial fibrillation. Int J Cardiol.

[119] Border WA, Noble NA (1994). Transforming growth factor beta in tissue fibrosis. N Engl J Med.

[120] Nakajima H, Nakajima HO, Salcher O (2000). Atrial but not ventricular fibrosis in mice expressing a mutant transforming growth factor-beta(1) transgene in the heart. Circ Res.

[121] Davies MJ, Pomerance A (1971). The morphological basis of atrial fibrillation in man. J Pathol.

[122] Thery C, Gosselin B, Lekieffre J, Warembourg H (1977). Pathology of
sinoatrial node. Correlations with electrocardiographic findings in
111 patients. Am Heart J.

[123] Hurlé A, Climent V, Sánchez-Quintana D (2006). Sinus node struc-tural changes in patients with long-standing chronic atrial fibrillation. J Thorac Cardiovasc Surg.

[124] Hurlé A, Sánchez-Quintana D, Ho SY (2010). Capillary supply to the sinus node in subjects with long-term atrial fibrillation. Ann Thorac Surg.

[125] Bruins P, te Velthuis H, Yazdanbakhsh AP (1997). Activation of the complement system during and after cardiopulmonary bypass surgery: postsurgery activation involves C-reactive protein and is associated with postoperative arrhythmia. Circulation.

[126] Gaudino M, Andreotti F, Zamparelli R (2003). The -174G/C interleukin-6 polymorphism influences postoperative interleukin-6 levels and postoperative atrial fibrillation: is atrial fibrillation an inflammatory complication?. Circulation.

[127] Spodick DH (1976). Arrhythmias during acute pericarditis: a prospective study of 100 consecutive cases. JAMA.

[128] Frustaci A, Chimenti C, Bellocci F (1997). Histological substrate of atrial biopsies in patients with lone atrial fibrillation. Circulation.

[129] Conway DS, Buggins P, Hughes E, Lip GY (2004). Relationship of interleukin-6 and C-reactive protein to the prothrombotic state in chronic atrial fibrillation. J Am Coll Cardiol.

[130] Aviles RJ, Martin DO, Apperson-Hansen C (2003). Inflammation as a risk factor for atrial fibrillation. Circulation.

[131] Chung MK, Martin DO, Sprecher D (2001). C-reactive protein elevation in patients with atrial arrhythmias: inflammatory mechanisms and persistence of atrial fibrillation. Circulation.

[132] Hack CE, Wolbink GJ, Schalkwijk C (1997). A role for secretory phospholipase A2 and C-reactive protein in the removal of injured cells. Immunol Today.

[133] Mihm MJ, Yu F, Carnes CA (2001). Impaired myofibrillar energetics and oxidative injury during human atrial fibrillation. Circulation.

